# Ozone and Wounding Stresses Differently Alter the Temporal Variation in Formylated Phloroglucinols in *Eucalyptus globulus* Leaves

**DOI:** 10.3390/metabo9030046

**Published:** 2019-03-06

**Authors:** Bin Liu, Bruna Marques dos Santos, Arooran Kanagendran, Elizabeth H. Jakobsen Neilson, Ülo Niinemets

**Affiliations:** 1 Chair of Crop Science and Plant Biology, Estonian University of Life Sciences, Kreutzwaldi 1, Tartu 51006, Estonia; kanagendran.arooran@unine.ch (A.K.); Ylo.Niinemets@emu.ee (Ü.N.); 2 Plant Biochemistry Laboratory, Department of Plant and Environmental Sciences, University of Copenhagen, Thorvaldsensvej 40, DK-1871 Frederiksberg C, Denmark; brms@plen.ku.dk (B.M.d.S.); en@plen.ku.dk (E.H.J.N.); 3 Institute of Biology, Faculty of Science, University of Neuchâtel, rue Emile-Argand 11, 2000 Neuchâtel, Switzerland; 4 Estonian Academy of Sciences, Kohtu 6, 10130 Tallinn, Estonia

**Keywords:** *Eucalyptus globulus*, formylated phloroglucinol compounds (FPCs), wounding, ozone, macrocarpal, sideroxylonal

## Abstract

Formylated phloroglucinol compounds (FPCs) are a class of plant specialized metabolite present in the Myrtaceae family, especially in the genus *Eucalyptus*. FPCs are widely investigated due to their herbivore deterrence properties and various bioactivities of pharmaceutical relevance. Despite the increasing number of studies elucidating new FPCs structures and bioactivity, little is known about the role of those compounds in planta, and the effects of environmental stresses on FPC concentration. Ozone (O_3_) and wounding are key stress factors regularly confronted by plants. In this study, we investigated how O_3_, wounding, and their combination affected individual and total FPC foliar concentration of the economically important species *Eucalyptus globulus*. Six individual FPCs, including five macrocarpals and one sideroxylonal, showed different response patterns to the single and combined stresses. Total macrocarpals only increased under single O_3_ treatment, whereas total sideroxylonals only increased in response to wounding treatment, suggesting different physiological roles played by the two groups of FPCs predominantly existing in *E. globulus* foliage. Total FPCs increased significantly under individual wounding and O_3_ treatments but not under the combined treatment. A principal component analysis indicated that all different treatments had unique FPC fingerprints. Total phenolic contents increased in all O_3_ and wounding treatments, and a marginally positive correlation was found between total FPCs and total phenolic contents. We suggest that, depending on the concentration and composition, FPCs play multiple physiological roles in planta, including serving as antioxidants to scavenge the reactive oxygen species brought about by O_3_ and wounding stresses.

## 1. Introduction

Ozone (O_3_) is considered as a key air pollutant formed in the troposphere by photochemical reactions between nitrogen oxides (NO_x_), and volatile organic compounds (VOCs) such as hydrocarbons in the presence of the sunlight [1,2]. Due to increased anthropogenic activities, the tropospheric O_3_ levels have increased by 30% since the pre-industrial era [3]. As a phytotoxic pollutant, ambient O_3_ can enter plants through the stomata and once inside the leaf, O_3_ will further lead to the endogenous formation of reactive oxygen species (ROS) and eventually trigger programmed cell death, particularly in O_3_-sensitive plant species [4,5,6]. Typically, high O_3_ concentrations are commonly applied as a tool to study the O_3_-induced ROS and signaling mechanisms [4,5,6,7].

Elevated O_3_ can cause acute morphological, physiological, and biochemical changes in plants. It can severely curb photosynthesis and other primary metabolic reactions and has an especially large effect on specialized plant metabolism, which plays an important role in directly and indirectly alleviating the damages caused by O_3_ [7,8,9,10,11,12,13,14]. For example, Kanagendran et al. [7] demonstrated that acute O_3_ fumigation induced a higher rate of isoprene, and strong mono- and sesquiterpene emissions associated with O_3_-scavenging mechanisms in *Eucalyptus globulus* leaves. In addition to changes in terpenoid metabolism, accumulation of phenolics including phenylpropanoids, flavonoids, tannins, and anthocyanins have been shown to be strongly enhanced by O_3_, and possibly involved in scavenging O_3_-induced ROS [11,15,16]. 

In the natural environment, plants frequently encounter multiple stress factors, including O_3_ stress, either simultaneously or sequentially, i.e., elevated O_3_ exposures, heavy precipitation, herbivore feeding, and acute heat episodes [17,18,19,20]. Wounding is a typical stress factor caused by both biotic (e.g., herbivore feeding) and abiotic (e.g., strong wind, heavy precipitations, and moving objects) agents in natural ecosystems [21,22,23]. To cope with the mechanical damages of wounding and prevent further pathogen infection or herbivore attack via the wounded tissues, plants often activate both constitutive and induced defense mechanisms, primarily involving massive production of specialized metabolites [23,24]. In fact, several reports have demonstrated the protective roles of phenolic compounds as a physical barrier and antimicrobial substances against abiotic and biotic stresses [22,23,25]. Among several groups of phenolic compounds, the formylated phloroglucinol compounds (FPCs) found in plants from the Myrtaceae family, primarily in *Eucalyptus* species have drawn attention due to their complex chemical structures and the efficient herbivore deterrence properties upon feeding of *Eucalyptus* leaves by folivores, especially for marsupials such as the iconic koala [26,27,28,29]. Under moderate environmental stresses, changes in photosynthesis and leaf water relations of eucalypt leaves can be minor, while changes in specialized metabolites constitute the main alterations in the phenotype [30,31,32]. 

FPCs are a group of specialized metabolites consisting of a phloroglucinol-based derivative often with an attached mono- or sesquiterpene moiety [26,33]. The simplest FPCs are fully substituted formylated acylphloroglucinols, such as jensenone. The units of jensenone form the basis of dimeric acylphloroglucinols, such as sideroxylonals A, B, and C. Sideroxylonals are compounds with a 2-phenylchromane skeleton, containing four formyl groups located in the aromatic rings at the positions C-3, C-5, C-3′, and C-5′, an isobutyl at C-7, and the isopropyl substituent at C-10′. The differences between individual sideroxylonals appear in the stereochemistry at C-7 and C-10’ [34]. The formylated acylphloroglucinols can also form adducts with mono- and sesquiterpenes, such as euglobals and macrocarpals [26,28]. The macrocarpals possess an unusual skeleton that can be divided into two domains—one domain comprising a phloroglucinol dialdehyde moiety (common to all macrocarpals) and a second terpenoid domain [35]. 

Due to the pharmaceutical and therapeutical values such as inhibiting the reverse transcriptase of HIV [36], antimicrobial, anticancer [37], and antimalarial effects, previous studies have mostly focused on elucidating the chemical structure of novel FPCs extracted from different tissues, such as bark, leaf, fruit, and wood [28,38,39]. The most studied role of FPCs in planta relates to herbivore deterrence, with significant ecological importance. For example, the concentration of total FPCs was the most important variable determining feeding by marsupial folivores on *Eucalyptus* species [40,41,42] and played an important role in determining habitat patchiness of Australian forests [41]. To a large extent, the limited information of the role of FPCs in planta has been due to challenges related to FPC identification and quantification, as the majority of FPCs occur as structurally similar isomers that are difficult to individually separate and distinguish between within a complex biological sample. Chemical synthesis of FPCs have been attempted [43], however, this is a difficult and costly process. Consequently, there is a limited number of commercially available authentic FPC standards, thereby constraining quantitative analysis of FPC levels in planta, such as measuring a differential response to various environmental conditions.

In this study, we used *Eucalyptus globulus* Labill. (Tasmanian blue gum), a fast-growing tree species from Southern Australia which is grown as a plantation tree in many warm temperate and subtropical regions all over the world. Its foliage is enriched with several groups of specialized metabolites—primarily terpenoids and FPCs—and it is considered as a promising species for pharmaceutical applications [44]. *Eucalyptus globulus* has been demonstrated to be a strong constitutive volatile terpenoid emitter [7,12,45]. In addition, the presence of different FPCs, including macrocarpals A, B, D, H, I, J, N, P, Q, and sideroxylonal A, have also been observed in *E. globulus* leaf, bark, and wood tissues [26,28,46,47]. 

In the present study, we obtained a number of the commercially available FPC standards to quantitatively investigate how acute O_3_ and wounding stress affect the individual and total FPCs concentrations in *E. globulus* foliage. Our previous studies demonstrated that O_3_ and wounding stress differentially regulate terpene synthase gene expression and terpene emission responses in a time-dependent manner [7,12]. Therefore, we hypothesized that (i) the O_3_ and wounding treatments alone, as well as in combination, will affect FPC composition in *E. globulus* leaf tissues, and (ii) due to the general antioxidant properties of phenolic compounds, both total phenolic and FPC concentration will increase through time in response to O_3_ and wounding treatments. The results obtained in this study provide evidence of coordinated variation of different metabolic pathways and as such provide novel information of how specialized metabolism is affected by abiotic stress in *Eucalyptus*.

## 2. Results

### 2.1. Wounding and O_3_ Treatments Alter Individual FPC Concentrations in E. globulus Leaves

**Figure 1:**
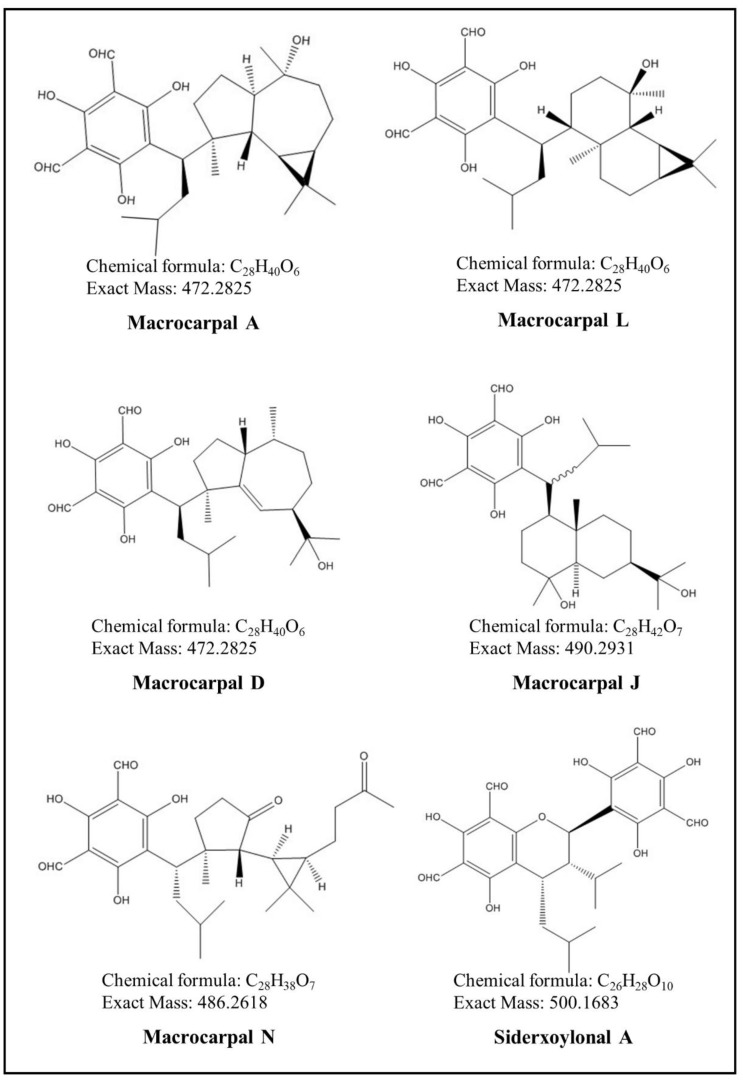
Structural formulas of six different FPCs identified in *E. globulus* leaves.

**Figure 2:**
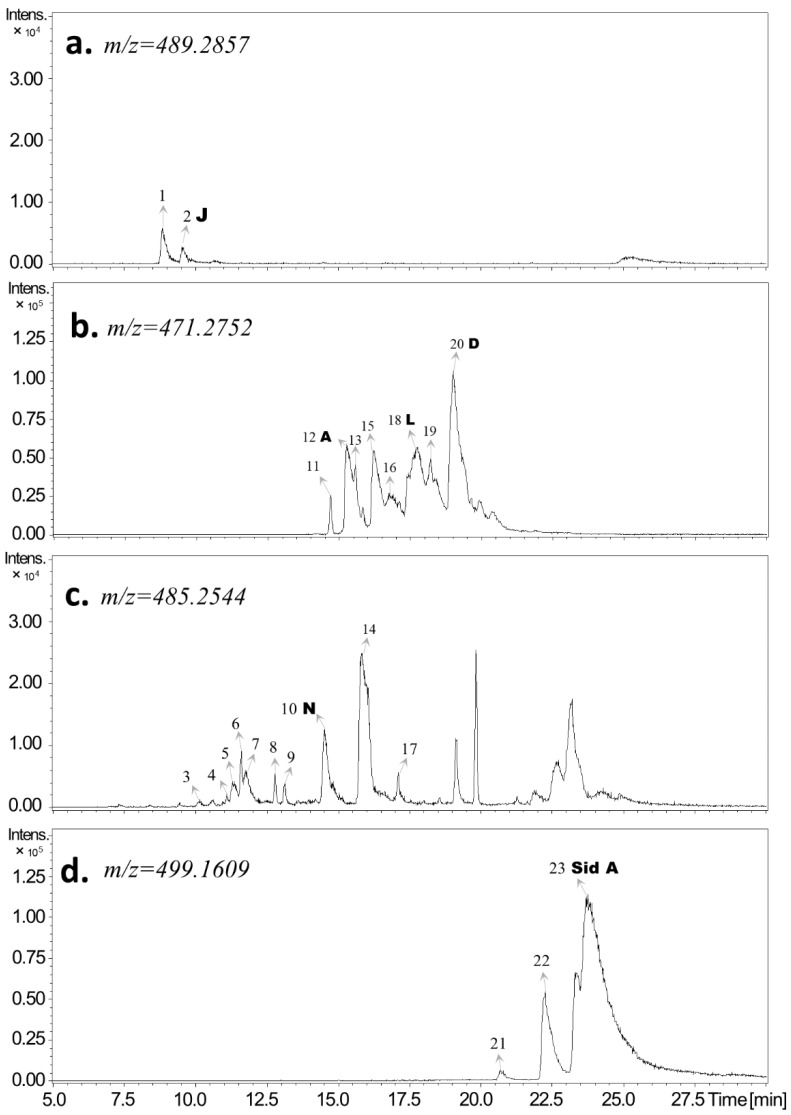
Representative UHPLC-DAD-qToF-MS/MS extracted ion chromatograms showing FPCs detected in *E. globulus* leaves. The FPCs detected in *E. globulus* leaves are divided into four groups according to *m*/*z* values [M−H]^−^ of 489.2857 (**a**), 471.2752 (**b**), 485.2544 (**c**), and 499.1609 (**d**). Based on co-elution with authentic standards, known FPCs are indicated by arrows with letters. See FPC identification in Material and Methods and the peak details in Table 1. Abbreviations: A, macrocarpal A; D, macrocarpal D; J, macrocarpal J; L, macrocarpal L; N, macrocarpal N; and Sid A, sideroxylonal A.

**Figure 3:**
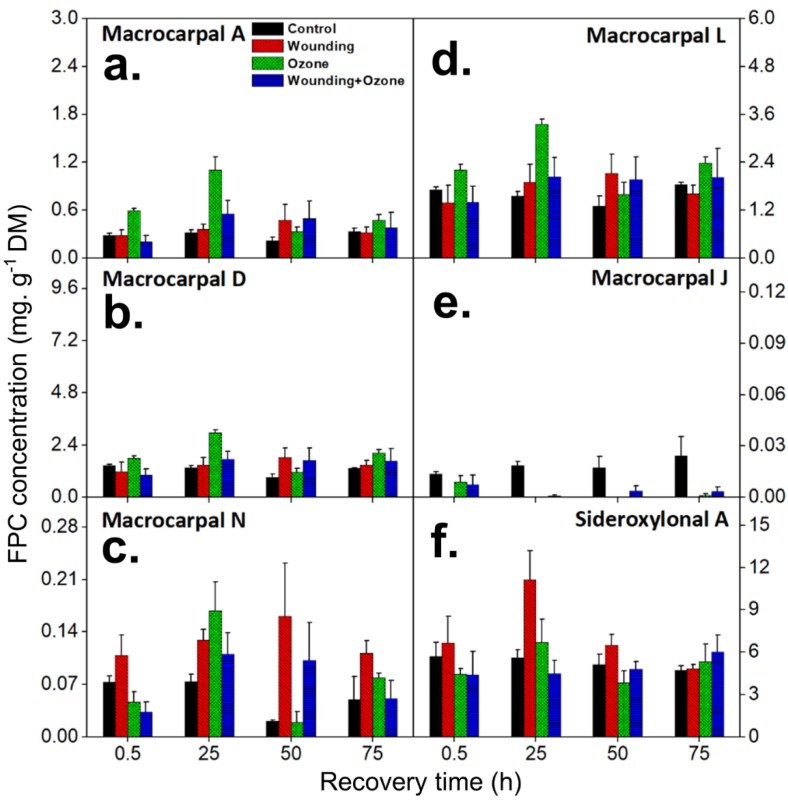
Temporal changes in individual FPCs (means ± SE, *n* = 3) in *E. globulus* leaves subjected to separate and combined ozone (O_3)_ and wounding treatments. Linear mixed models were applied to analyze the fixed effects of different treatments (wounding, O_3_, and O_3_ + wounding) and recovery times on the temporal changes in FPCs concentrations in *E. globulus* leaves. The summary of the linear mixed model is shown in Table 2.

In this study, the detected FPCs were classified into two main groups of FPCs—macrocarpals and sideroxylonals (herein defined as total FPCs; see material and methods)—and analyzed by the UHPLC-DAD-ESI-Q-TOF-MS/MS, combining parental ion accurate mass and diagnostic MS2 daughter ions (Table 1). Individual FPCs macrocarpal A, macrocarpal D, macrocarpal N, macrocarpal L, macrocarpal J, and sideroxylonal A were identified and quantified using authentic standards (Figure 1 and Figure 2). Among these six FPCs, sideroxylonal A was the most abundant compound in untreated leaf tissue, ranging from 4.72 ± 0.32 to 5.70 ± 0.97 mg g^−1^ DM, while the macrocarpal J was the least abundant compound with only 0.024 ± 0.011 mg g^−1^ DM (Figure 3). Wounding alone significantly affected the concentrations of macrocarpal L, macrocarpal N, macrocarpal J, and sideroxylonal A in *E. globulus* leaves (Figure 3 and Table 2). Both macrocarpal L and macrocarpal N reached the peak concentrations at 50 h, increasing approximately 2-fold to 2.12 ± 0.49 mg g^−1^ DM and 0.16 ± 0.07 mg g^−1^ DM, respectively in the wounded leaf tissue compared to control leaves (Figure 3c,d). In contrast, wounding resulted in decreased macrocarpal J beyond the detection limit at all recovery times (Figure 3e). Compared to the time taken for macrocarpal L and N to reach the maximum, sideroxylonal A was highly responsive for wounding treatment alone, as the concentration maximum of 11.1 ± 2.1 mg g^−1^ DM was observed at 25 h after the wounding treatment (Figure 3f). This maximal accumulation also made sideroxylonal A the most abundant FPC among all the detected compounds in wounded *E. globulus* leaves (Figure 2 and Figure 3f).

Ozone fumigation significantly affected the concentrations of all six identified FPCs (Table 2). The concentrations of macrocarpal A, macrocarpal D, macrocarpal N, and macrocarpal L all increased significantly in *E. globulus* leaves compared to controls (Figure 3a–d and Table 2). Of these four FPCs, macrocarpal A showed the fastest response to O_3_ treatment, significantly increasing to 0.60 ± 0.03 mg g^−1^ DM at 0.5 h after O_3_ treatment, more than 2-fold higher than control leaves (Figure 3a). In contrast to the wounding treatment where maximum macrocarpal response occurred at 50 h, O_3_ treatment induced an earlier response of macrocarpal A, D, N, and L, with maximum concentration measured after 25 h. For example, at 25 h, macrocarpal A concentration reached up to 1.12 ± 0.17 mg g^−1^ DM, more than a 3-fold increase compared to control leaves (Figure 3a). Similarly, peak concentrations of macrocarpals D, N, and L significantly increased more than 2-fold, reaching 2.95 ± 0.14 mg g^−1^ DM, 0.17 ± 0.037 mg g^−1^ DM, and 3.35 ± 0.14 mg g^−1^ DM, at 25 h of recovery, respectively (Figure 3b–d). In response to O_3_ treatment, macrocarpal J concentrations decreased at 0.5 h of recovery and were generally below the levels of detection during the recovery period thereafter (Figure 3e). Sideroxylonal A also increased to its maximum concentration of 6.67 ± 1.65 mg g^−1^ DM (1.2-fold of controls) at 25 h after the O_3_ treatment, but otherwise remained at lower or similar concentrations to control levels during the recovery period (Table 2 and Figure 3f).

Combined treatments of O_3_ and wounding (O_3_ + wounding) affected the concentrations of three FPCs—macrocarpal A, macrocarpal N, and sideroxylonal A (Table 2). The O_3_ + wounding treatments led to a significant increase of both macrocarpal A and macrocarpal N (Figure 3a,c). At 25 h of recovery after combined treatments of O_3_ + wounding, the concentrations of macrocarpal A and macrocarpal N reached concentration maxima of 0.55 ± 0.17 mg g^−1^ DM and 0.11 ± 0.03 mg g^−1^ DM, respectively. In contrast, sideroxylonal A concentrations exhibited a rapid and significant decrease at 0.5 h, to approximately 77% of the control levels. During the recovery period, sideroxylonal A concentrations gradually recovered to reach the control level at the end of the recovery phase (Figure 3f). Notably, in contrast to the individual wounding and O_3_ treatments, combined O_3_ + wounding did not significantly affect macrocarpal J concentrations (Figure 3e).

**Figure 4:**
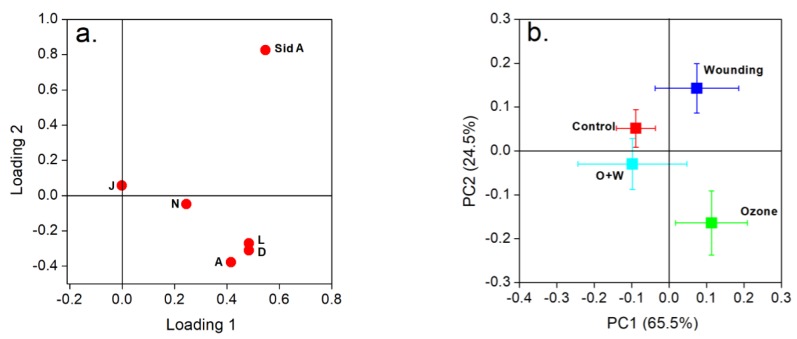
Principal component analysis (PCA) of FPCs as affected by wounding and O_3_ treatments in *E. globulus* leaves—loading (**a**) and score plot (**b**). Individual FPCs concentrations across the recovery period were used in this analysis. The O + W in (b) corresponds to the combined O_3_ and wounding treatment. Abbreviations: A, macrocarpal A; D, macrocarpal D; J, macrocarpal J; L, macrocarpal L; N, macrocarpal N; and Sid A, sideroxylonal A.

The principal component analysis (PCA) indicated that the first two principal components (PC1, 65.5% and PC2, 24.5%) described 90.0% of the variations among the concentrations of six individual FPCs in *E. globulus* leaves across different treatments and controls (Figure 4). The distribution of individual FPCs in the PCA loading plot (Figure 4a) and treatment clusters in the PCA score plot (Figure 4b) showed clear variation in individual FPC accumulation in controls, and in the separate and combined O_3_ and wounding treatments. The wounded leaves were distinctly differentiated from controls and all other treatments due to the increased accumulation of sideroxylonal A. In addition, changes in macrocarpal J concentration clearly differentiated controls from all treated leaves. Furthermore, separate O_3_ treatments led to stronger correlations among the concentrations of macrocarpal A, macrocarpal L, and macrocarpal D (Figure 4a,b). Overall, the strongest correlations were observed between the changes in macrocarpal L and macrocarpal D in leaves at different recovery times (Figure 4a,b). PCA further confirmed that the changes in foliar macrocarpal A, macrocarpal L, and macrocarpal D upon O_3_ exposure alone were almost identical throughout the recovery phase (Figure 3 and Figure 4a,b). 

### 2.2. Wounding and O_3_ Treatments Affect Total FPC and Phenolic Content in E. globulus Leaves

**Figure 5:**
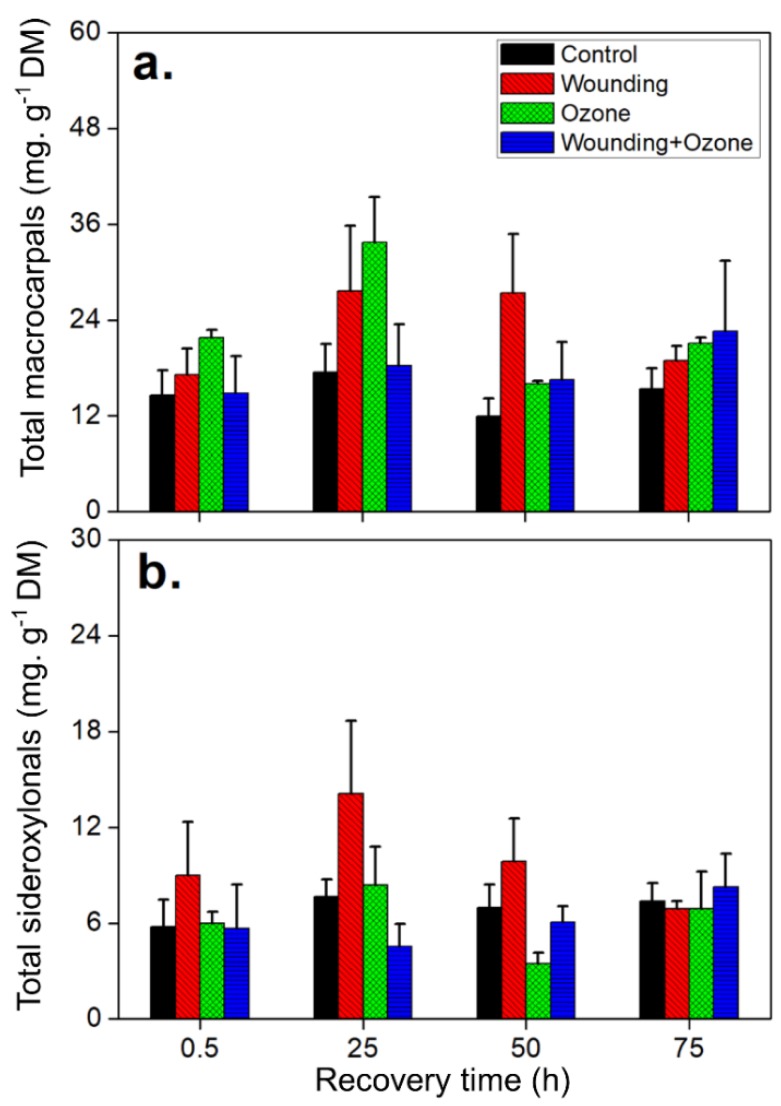
Time-dependent changes (means ± SE, *n* = 3) in the concentration of total macrocarpals (**a**) and total sideroxylonals (**b**) in *E. globulus* leaves subjected to separate and combined wounding and O_3_ treatments. Details of the statistical summary are shown in Table 3. Total macrocarpals are expressed as mg g^−1^ DM macrocarpal A equivalents, and total sideroxylonals are expressed as mg g^−1^ DM sideroxylonal A equivalents.

In addition to the six individual FPCs identified, total FPCs and phenolics were measured. Specifically, total FPCs were measured by combining total macrocarpals and total sideroxylonals identified by UHPLC-DAD-ESI-Q-TOF-MS/MS analysis. The wounding treatment only showed significant impacts on total sideroxylonals compared to total macrocarpals (Table 3 and Figure 5). A clear increasing trend was observed in total macrocarpals in response to wounding. Total macrocarpal concentrations increased to 27.4 ± 7.4 mg g^−1^ DM (more than a 2-fold increase compared to control leaves) although this increase was only marginally significant (*F* = 4.42, *Sig.* = 0.08; Table 3 and Figure 5a). Notably, the concentration of total sideroxylonals in the wounded leaves was higher than that in O_3_ treated leaves, particularly at 25 and 50 h after the treatments (Figure 5b). Separate O_3_ treatments resulted in the greatest enhancement of total macrocarpals in *E. globulus* leaves, with the concentrations rising up to 33.7 ± 5.7 mg g^−1^ DM, compared to 17.5 ± 3.6 mg g^−1^ DM in control plants at 25 h after treatments (Figure 5a). In contrast, the combined O_3_ + wounding treatments only affected the foliar contents of total sideroxylonals, with a significant decrease in concentration (Table 3 and Figure 5b).

**Figure 6:**
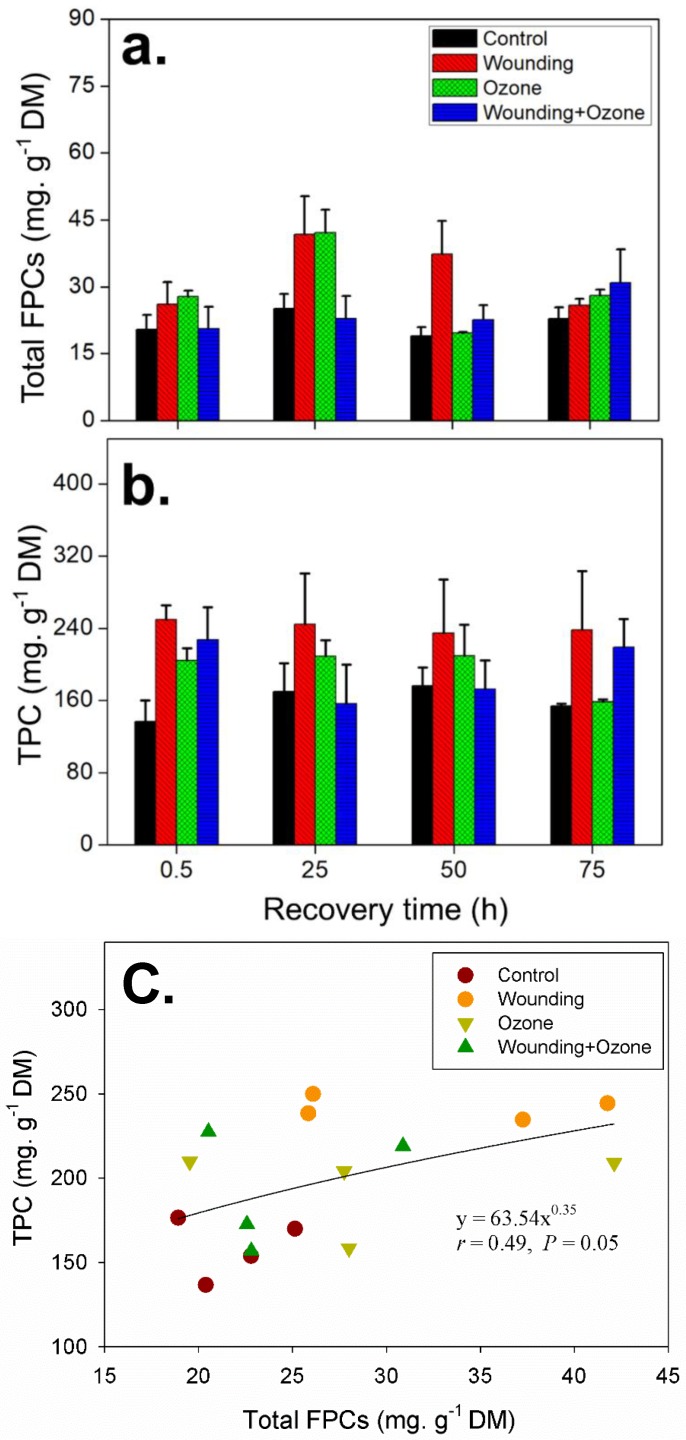
Temporal changes (mean ± SE, *n* = 3) in the concentration of total FPCs (**a**), total phenolic compounds (**b**), and their correlation (**c**) in *E. globulus* leaves subjected to separate and combined O_3_ and wounding treatments. The data selected for non-linear correlation analysis are the mean of total FPCs and total phenolics concentrations at each recovery time point from different treatments and controls. Total phenolic contents (TPC) are expressed as mg g^−1^ DM gallic acid equivalents. Details of the statistical summary are shown in Table 3.

The concentration of total FPCs was more strongly affected by separate wounding and O_3_ treatments than by combined O_3_ and wounding treatment (Figure 6a and Table 3). The total FPCs concentration peaked at 25 h after treatment for wounding (total FPC concentration = 41.8 ± 8.5 mg g^−1^ DM) and for O_3_ (FPC concentration = 42.1 ± 5.2 mg g^−1^ DM) (Figure 6a). After 25 h, the total FPCs decreased to pre-stress level (18.9 ± 2.0 mg g^−1^ DM) under O_3_ treatment, whereas the total FPC in wounded leaves was still maintained at a significantly higher concentration (Figure 6a). 

Total phenolic content was significantly affected by all separate and combined O_3_ + wounding treatments (Figure 6b and Table 3). In the wounded leaves, total phenolic content increased up to 2-fold compared with controls, reaching 250 ± 15 mg g^−1^ DM at 0.5 h. Thereafter, total phenolic content decreased to 238 ± 65 mg g^−1^ DM at 75 h after the treatment but remained more than 1.5-fold higher than control levels (Figure 6b). Similarly, O_3_ treatments resulted in significant increases in the accumulation of total phenolics until 50 h after the treatment but recovered to control levels at 75 h (Figure 6b). When subjected to a combined O_3_ and wounding treatment, increases in total phenolic content were only measured at 0.5 h (1.7-fold) and 75 h (1.4-fold) during the recovery period (Figure 6b). A marginally significant positive correlation (*r* = 0.49; *P* = 0.05) was observed between the total FPCs and total phenolic contents when pooling the data from all the treatments (Figure 6c).

### 2.3. Wounding and O_3_ Treatments Alter the FPC Composition in E. globulus Leaves

The composition of individual FPCs was significantly altered under different treatments (Table 4 and Table 5). Of the six FPCs identified in this study, the order of their proportions of total FPCs in control leaves was sideroxylonal A (25.3 ± 4.4%), macrocarpal L (7.7 ± 0.6%), macrocarpal D (5.99 ± 0.49%), macrocarpal A (1.33 ± 0.10%), macrocarpal N (0.240 ± 0.042%), and macrocarpal J (0.080 ± 0.015%). However, wounding and O_3_ treatments performed time-dependent effects on the composition of some compounds, e.g., macrocarpal A, macrocarpal D, macrocarpal N, and sideroxylonal A (Table 4). Generally, sideroxylonal A was the most abundant and macrocarpal J was least abundant among the six individual FPCs. In particular, except for macrocarpal N and sideroxylonal A, wounding alone led to general decreases in all other individual FPCs. Meanwhile, separate O_3_ and combined O_3_ and wounding treatments resulted in an overall enhancement of most individual macrocarpals, but the highest levels were observed at different recovery times for different treatments (Table 4 and Table 5). However, the proportion of macrocarpal J decreased to nearly zero in leaves under all treatments compared to control, and the concentration of macrocarpal J in controls was very low in all cases (Table 5 and Figure 3e).

## 3. Discussion

FPCs constitute an interesting and valuable group of specialized metabolites in *Eucalyptus* trees. In this study, we investigated the effects of single and combined O_3_ and wounding stresses on the six selected FPCs present in *E. globulus* foliage [26,28,46,47]. Concentrations of different identified FPCs are highly variable in *E. globulus* leaves (Figure 2 and Figure 3) and have been reported previously by Lawler et al. [41]. The stress-dependent changes in concentrations of different individual FPCs observed in our study can provide illuminating insight into their potential physiological functions in stressed leaf tissues. Taking macrocarpals as examples, the concentration of macrocarpal D was only impacted by O_3_ treatment, whereas the other macrocarpals were affected by either separate or combined application of O_3_ and wounding treatments (Figure 3 and Table 2). Considering that there are very few studies focusing on FPCs response to environmental stresses, the results presented here will improve our understandings of the possible role of individual FPCs in plant defense mechanisms. 

### 3.1. FPCs Responses to O_3_ Stress

Depending on the concentrations, the exposure to elevated O_3_ leads to chronic or acute damages by first affecting the photosynthesis apparatus in exposed leaf tissues [48,49]. Compared with many other species, *E. globulus* has been proved to be an O_3_-tolerant tree species to both long- and short-term O_3_ treatments. For example, long-term elevated O_3_ fumigation resulted in no visible injury or dry-weight reduction in *E. globulus* [50]. Our recent study also showed that the photosynthetic characteristics were resistant to the acute O_3_ fumigation in *E. globulus* leaves [7]. Emissions of volatile terpenoids, such as isoprene, monoterpenes, and sesquiterpenes, are considered to play an important role to quench O_3_ oxidation in the very early stage of O_3_ stress [7,49,51]. Volatiles can play an antioxidant role primarily in the lipid phase or the gas phase (e.g., reducing potentially damaging volatile ROS effects), whereas condensation with phloroglucinols makes them non-volatile, suggesting that some FPCs could have antioxidant properties in lipid and liquid phases. To our knowledge, no studies have confirmed antioxidant properties of FPCs in *Eucalyptus* trees. The terpene moiety in some of the FPC structures suggests a possible antioxidant potential, and the aromatic phloroglucinol moiety is likely to have antioxidant properties as well [52,53]. Indeed, in the current study, we found a clear increasing trend from both individual and total FPCs across the recovery period in response to different stress applications (Figure 3 and Figure 6). This predominant increase, however, is mainly occurring in late recovery phases, particularly after 25 h of O_3_ treatment (Figure 3 and Figure 5). In contrast, terpenoid emission bursts occurred in early recovery phase applications, especially after 0.5–10 h of treatments in *E. globulus* leaves [7]. 

Interestingly, macrocarpals displayed higher sensitivity to O_3_ stress than sideroxylonals. It was further evident in our study that separate O_3_ treatment did not have a large impact on the concentration of total sideroxylonals, but separate O_3_ treatment substantially influenced total macrocarpal concentrations (Table 3). Furthermore, the proportion of macrocarpals and sideroxylonals in total FPCs was changed in O_3_ stressed leaves in a time-dependent manner (Table 4 and Table 5). We presume that the structural differences between macrocarpals and sideroxylonals might have resulted in such a different permanence under O_3_ oxidative stress. In fact, higher lipid solubility conferred by the terpene moiety allows the macrocarpals to carry the reactive aldehyde and phenol groups across the membranes more easily [54]. Structurally, macrocarpals and sideroxylonals have the same acylphloroglucinol skeleton; however, macrocarpals have a free hydroxyl, whereas the sideroxylonals have an ether linkage connecting another phloroglucinol unit (Figure 1). This suggests that macrocarpals will be able to contribute significantly to antioxidant properties in the lipid phase, due to their higher membrane permeability. Moreover, due to the direct antioxidant property of terpenoids [49,51,55], it is more likely that macrocarpals with the terpene moieties also own similar antioxidant properties as for terpenoids. The direct antioxidant properties of macrocarpals deserve future studies. 

As antioxidants, total phenolics content usually significantly increase in response to O_3_ stress in many plant species [15,16,56]. Consistently under O_3_ stress, we also observe such significant increases of total foliar phenolics as early as 0.5 h after the O_3_ treatments (Figure 6b), indicating a quick response of *E. globulus* leaves to O_3_ stress. However, different from total FPCs which have a peak concentration at 25 h, the total phenolic contents remained at a high level until 50 h and dropped to the control level 75 h after separate O_3_ treatment (Figure 6b). Furthermore, previous reports indicated that O_3_ treatment can quickly induce phenylalanine ammonia-lyase (PAL) activity in leaf tissues [57,58,59]. For example, the PAL activity concomitant with individual phenolic compounds including caffeic acid, ferulic acid, and catechin significantly increased 2 h after the O_3_ exposure in *Vitis vinifera* L. leaves [58]. If the FPCs have antioxidant properties, as presumed before, they might not play a role in an early stage of the O_3_ stresses; but rather, other metabolites, including constitutive and induced terpenoids and other phenolic compounds, are involved in early oxidation resistance [7,14,58].

### 3.2. FPCs Responses to Wounding Stress

Wounding can occur instantaneously by herbivore feeding and other mechanical damages, such as those caused by wind. In response to wounding stress, plants instantly release volatile constitutive specialized metabolites such as mono- and sesquiterpenes, particularly those stored in the wounded tissues [7,60]. Despite FPCs being constitutively stored in the *E. globulus* foliage, we did not observe significant increases of individual FPCs in a short time (0.5 h) as the volatile terpenoid emission bursts happened. Here, the majority of individual FPCs significantly increased at least 25 h after the wounding treatment (Figure 3), which suggests that FPCs are involved in a cascade of defense mechanisms against wounding stress. In particular, the concentration of the most abundant FPC, sideroxylonal A, increased in the wounded leaves to levels nearly twice those in the controls (Figure 3f). In this study, we further found that O_3,_ or its combination with wounding stress, can reduce sideroxylonal A concentration in *E. globulus* foliage, suggesting different physiological roles of sideroxylonal A in wounding and O_3_ stresses (Figure 3f and Table 2). This increase of sideroxylonal A in wounded leaf tissues is in agreement with the effective antifeedant role sideroxylonals possess towards marsupials and insects [28,61,62,63]. The increase in sideroxylonal A and other sideroxylonals was only sustained for 50 h before returning to control levels in wounded leaves (Figure 3f and Figure 5b, and Table 3). We, therefore, speculate that the subsequent deterrent effect after herbivore wounding, if any, might not depend on sideroxylonals alone but may operate in combination with FPC classes, such as macrocarpals. For example, following the wounding treatment, macrocarpal N showed a consistent higher concentration until the end of this experiment (Figure 3c), suggesting a possible deterrent role in a long-term herbivory response in *E. globulus*. In fact, complex mixtures of FPCs found in many trees provide an equivalent level of defense to that afforded by the same concentrations of single FPCs [42].

Among the six individual FPCs identified, macrocarpal J was the only compound to significantly decrease under all treatments (Figure 3e). The physiological function of this specific FPC is not known yet. Given a lack of antibacterial activity detected from macrocarpal J [47], together with a relatively low concentration (0.01–0.02 mg g^−1^ DM) and proportion (0.06%–0.09% of total FPCs) in this study (Figure 3e and Table 5), we infer that macrocarpal J may not play an essential role in wounding and O_3_ stress resistance in *E. globulus* foliage. Similarly, drought stress also led to an approximate 11% decrease of macrocarpal A and G in *E. globulus* leaf tissues, further indicating various physiological roles and response patterns of individual FPCs in plant stress resistance mechanisms [31].

In *E. globulus* wood tissues, macrocarpals were found to be present in higher concentrations compared to sideroxylonals [38]. This is also the case in the leaf tissues as we found in this study, which shows that the concentration of total macrocarpals is more than twice that of the total sideroxylonals (Figure 5). The total FPCs in untreated leaves of this study are comparable to those in previous studies, ranging from 20–30 mg g^−1^ DM [64]. Once wounded, the total foliar FPCs can significantly increase up to 45 mg g^−1^ DM (Figure 6a), indicating FPCs positively contribute to the environmental stress resistance, e.g., deterring herbivores from entering plants via the open wounds [38]. As expected, the total phenolic content significantly increased in wounded leaves and is a widely reported response in many other plant species subjected to wounding treatments [65,66]. Here, however, we observed that the increase of total FPCs was not regulated in a synchronous pattern with the total phenolic content, with maximum total FPCs occurring between 25–50 h whilst total phenolics remained at a significantly high level from 0.5 h following the wounding treatment (Figure 6 and Table 3). This finding indicates that other phenolic classes beyond FPCs play an active role in the wounding stress response during the initial stages of recovery. The phenylpropanoids and their derivatives from shikimate and downstream pathways might partly account for the immediate increase in total phenolics, as instantaneous wounding stress can enhance the gene expression for PAL [67]. For example, two flavonoids—luteolin and apigenin—were found to increase in 1 h in wounded liverwort *Marchantia polymorpha* plants [68]. 

### 3.3. FPCs Responses to Combination of O_3_ and Wounding

The consequence of simultaneously implementing several stress treatments can exacerbate the simple additive of single stresses on plants due to so-called “synergistic effects” [69,70]. However, the application of sequential stress treatment can result in a markedly different physiological plant response due to a priming stimulus activated by the previous treatment [71]. In our current study, no such “synergistic effects” were observed that different from single O_3_ or wounding treatments, combined O_3_ + wounding did not affect the total FPCs despite some impacts on individual FPCs (Table 3). In contrast, we have demonstrated that there were synergistic effects of sequential O_3_ and wounding treatments on terpenoid emissions from *E. globulus* foliage, which showed significantly higher emission rates of monoterpenes and sesquiterpenes than from any single treatments [7]. It cannot be ruled out that the combined stresses were too severe and suppressed both the physiological activities, including photosynthesis and the specialized metabolic activities [18]. If this is not the case, other physiological functions of FPCs should be further considered. For example, in this study, we found that O_3_ fumigation can induce more macrocarpal rather than sideroxylonal accumulations in *E. globulus* leaves (Figure 5 and Table 3). Therefore, it can be inferred that the biosynthesis of macrocarpals is induced and accumulated in response to the initial O_3_ stress and before the subsequent wounding treatment. If antioxidant properties exist for macrocarpals, these compounds would be further consumed by scavenging ROS raised by the following wounding treatment [72]. Although the subsequent wounding treatment can significantly induce sideroxylonal accumulation, this group of FPCs is only less than one third of the total FPCs in *E. globulus* foliage [28], which eventually results in unaffected concentrations of total FPCs in *E. globulus* foliage subjected to combined O_3_ and wounding stresses (Figure 4, Figure 5 and Figure 6 and Table 3).

### 3.4. Correlations of FPCs with Other Specialized Metabolites

Several biosynthetic pathways for FPCs have been proposed [37,73,74,75,76,77,78], but the biosynthesis has not yet been resolved. Due to the existence of a monoterpene or sesquiterpene moiety as part of the FPCs, particularly those found in *E. globulus* foliage, pathways responsible for the synthesis of terpenoids and phenolics are expected to be correlated in *Eucalyptus* species [79,80]. Moore et al. [28] found species–specific positive correlations between terpene and FPC concentrations. The results of their study confirm the possibility of dominant volatile monoterpenes acting as cues to folivores feeding on eucalypts containing complex mixtures of FPCs. Besides this, very few studies simultaneously focus on both terpenoid syntheses from the mevalonate (MVA) pathway or the 2-C-methyl-D-erythritol 4-phosphate/1-deoxy-D-xylulose 5-phosphate (MEP/DOXP) pathway, and phenolic syntheses from the shikimate/phenylpropanoid pathway as targets to explore plant resistance mechanisms against biotic and abiotic stresses [81,82]. Previously, we already found O_3_ and wounding resulted in emission bursts of mono- and sesquiterpenes in *E. globulus* leaves [7]. The current investigation on FPCs in *E. globulus* leaves further contributed to understanding the connections between terpenoids and phenolics under O_3_ and wounding stresses.

In our study, there was a weak positive correlation (*r* = 0.49, *P* = 0.05) between the concentrations of total FPC and total phenolic contents under all treatments throughout the recovery phase (Figure 6c). It should be noted that the total FPCs contribute very little to the total phenolic contents measured here, as the total phenolics assay determines polar phenolic content, and the majority of FPCs are nonpolar [41]. Accordingly, in another study on *Eucalyptus nitens*, total FPCs were demonstrated to comprise less than 1% of the total phenolics [83]. This positive correlation between total FPCs and total phenolics in our study further indicated a coherent stress response from both FPCs and other groups of phenolics, such as phenylpropanoids and flavonoids, in *E. globulus* foliage under oxidative stress.

To date, only macrocarpal C has been chemically synthesized by adding bicyclogermacrene to the benzyl cation in the laboratory [84]. In plants, immediate cross-talks between different metabolic pathways for macrocarpal biosynthesis can be identified by analyzing the terpene residues in their chemical structures (Figure 1). For example, globulol is structurally identical to terpene moiety of macrocarpal A; whereas α-, β-, or γ-eudesmol is connected to macrocarpal J which has a eudesmane-type skeleton (Figure 1; [28]). Furthermore, in FPCs, the terpene moieties, e.g., C5- in jensenone, C10- in euglobal Ia, or C15- in macrocarpal A, can be possibly derived from isopentenyl pyrophosphate (IDP) via the terpenoid pathway, namely the MVA pathway in cytosol and the MEP/DOXP pathway in plastids. If this is the case, competitions between terpenoid emissions and FPC syntheses will be highly anticipated to exist in stressed *E. globulus* foliage. Our previous study demonstrated that both monoterpenes and sesquiterpenes increased significantly as soon as the plants were subjected to O_3_ and wounding stresses, in which the emissions of 1,8-cineole, α-pinene, limonene, aromadendrene, α-gurjenene, and viridiflorene are the most abundant compounds representing more than 50% of total monoterpene and sesquiterpene emissions [7]. To explore the relationships between terpenoids and FPCs, we correlated the total FPC concentrations with emission rates of isoprene and the aforementioned individual and total mono-/sesquiterpenes under stresses (Figures S1–S3). Surprisingly, we found negative correlations between terpenoid emissions and FPC concentrations, which contrasts to previous studies that demonstrated positive correlations between concentrations of total FPCs and terpenoids in *Eucalyptus* leaf extracts [28,41]. Such negatively correlated FPC concentrations and terpenoid emissions can be explained by the different response times of the two groups of metabolites in stressed leaf tissues, and a considerable amount of terpenoids are removed due to the reaction with ROS formed upon O_3_ and wounding treatments [7]. Furthermore, this study and our previous study [7] indicated that a quick emission response of terpenoids compounds, together with the later accumulation of FPCs, built up a comprehensive and efficient stress-resistance mechanism in *E. globulus* leaves against oxidative stress. In addition, it should be noted that emitted volatile terpenoids do not necessarily reflect the current in vivo terpenoid concentrations, as emitted terpenoids generally represent a relatively small portion from the in vivo terpenoid pools. For example, the emission rate of 1,8-cineole is approximately 30 µg g^−1^ DM h^−1^, but the concentration of 1,8-cineole can reach 300 mg g^−1^ DM in storage pools of *E. globulus* leaves [7,28]. If the FPCs in stressed leaves were also positively correlated with the non-emitted in vivo terpenoids [28], it could be suggested that in a long-term recovery period, more carbon will be allocated to the synthesis of FPCs rather than terpenoids to cope with the stresses brought by O_3_ and wounding. At the current stage, our results provide the first hint that competitions might exist for the biosynthesis of FPCs and terpenoids in *E. globulus* leaves which can be further enhanced by environmental stresses such as O_3_ and wounding.

## 4. Material and Methods

### 4.1. Plant Materials

The present experiments were performed with 1-year-old *E. globulus* seedlings which were grown from seeds (OMC seeds Ltd., Lithuania) at the Estonian University of Life Sciences as described by Kanagendran et al. [7]. In short, the seeds of *E. globulus* were sown in 5 L pots filled with 1:1 mixed quartz sand (AS Silikaat, Tallinn, Estonia) and commercial garden soil (N:P:K=10:3:20, Kekkilä Group, Vantaa, Finland). The seedlings were grown for three weeks in a growth chamber (Percival AR-95 HIL, CLF Plant Climatics GmbH, Wertingen, Germany) under 12 h light period with the light intensity of 400–500 µmol m^−2^ s^−1^ at leaf surface, day and night temperatures of 28/25 °C, ambient CO_2_ concentration of 380–400 ppm, and relative air humidity of 60%–70%. The 3-week-old seedlings were transplanted into 10 L pots filled with the same soil and grown under similar growth conditions in a plant growth room until the completion of the experiments. Plants were watered every two days to soil field capacity and fertilized once a week with a liquid fertilizer containing macronutrients N, P, and K (5:5:6), and micronutrients B (0.01%), Cu (0.03%), Fe (0.06%), Mn (0.028%), and Zn (0.007%) (Baltic Agro, Lithuania).

### 4.2. Wounding and O_3_ Treatments

In this study, four different sets of experiments, i.e., (i) control (no treatments), (ii) wounding (7 cm length of cut on leaf), (iii) acute O_3_ exposure (5 ppm), and iv) acute O_3_ exposure (5 ppm) followed by wounding were implemented as described by Kanagendran et al. [7]. A custom-made chamber system [85] was used for O_3_ fumigation and volatile collection from control and treated leaf tissues. In this system, the chamber temperature was maintained at 25 °C (leaf temperature at 25–27 °C) by circulating water between the double layers of the glass chamber. Light intensity at the leaf surface in the chamber was kept at 700–750 µmol m^−2^ s^−1^ by four 50 W halogen lamps above the chamber. Chamber CO_2_ concentration was 380–400 ppm and relative air humidity 60%–70%. Ambient air was constantly pumped through a charcoal-filled filter and a custom-made O_3_ trap (passing less than 2 ppb O_3_) before going into the glass chamber. An ozonizer (Certizon C100, Erwin Sander Elektroapparatenbau GmbH, Germany) was connected to the chamber inlet to provide O_3_ as required. O_3_ concentrations in ingoing and outgoing air streams were monitored by a UV photometric O_3_ detector (Model-49i, Thermo Fisher Scientific, Franklin MA, USA). 

A previous report indicated that continuous exposure of 0.8 ppm O_3_ for 6 h did not even cause physiological disturbances in *E. globulus* leaves and therefore, *E. globulus* are categorized as “extremely resistant” plant species to acute O_3_ exposures [86]. In addition, a preliminary study by Kanagendran et al. [7] found that exposure of *E. globulus* leaves to 0.3–2 ppm O_3_ did not cause a considerable change in photosynthetic characteristics and lipoxygenase (LOX) pathway emission rates. Therefore, we used higher acute O_3_ exposures in this study. In fact, we observed that O_3_ stress influences plants in an O_3_-dose (O_3_-sum) dependent manner that is dependent on both the concentration and the duration of O_3_ exposures (O_3_ concentration above a threshold limit × exposure time) [7,12,87]. However, as chronic O_3_ stress impacts plant physiological activity quite differently due to secondary acclimation responses [87,88], we consider the higher acute O_3_ exposures as a representative for quantitative characterization of plant responses to different O_3_ stress levels [7,12,89].

In each experiment, a randomly selected branch consisting of six fully mature leaves was used. For the wounding treatment, four 25 mm^2^ holes (7 cm of total perimeter length cut) were rapidly (within 6 s) made in each leaf lamina by a paper punch. In the case of O_3_ treatments, a branch consisting of 6 leaves was exposed to 5 ppm O_3_ for 3 h. For the combination of O_3_ and wounding treatments, first 5 ppm O_3_ was applied for 3 h, and then the 4 holes were punched in leaf laminas as soon as O_3_ exposure was terminated. The fresh leaf material was collected at 0.5, 25, 50, and 75 h after each treatment and immediately frozen in liquid nitrogen and then, homogenized with a mortar and a pestle. The homogenized leaf tissue was stored at −80 °C for the analysis of FPCs and total phenolics. 

### 4.3. FPCs Extraction and UHPLC-DAD-ESI-Q-TOF-MS/MS Analysis

The extraction, detection, and quantification of FPCs were performed according to Santos et al. [90]. Briefly, FPCs were extracted from approximately 100 mg of frozen and homogenized plant material. Then, 5 µL of the extracts were separated using a Phenomenex Kinetex^®^ column (150 × 2.1 mm) packed with 1.7 μm C18 material with a pore size of 100 Å. Extracts were eluted at a constant flow rate of 0.3 mL min^−1^ as follows: 50% solvent A (0.05% formic acid in water) and 50% solvent B (0.05% formic acid in acetonitrile) linearly increasing to 100% solvent B in 20 min, followed by hold time for 13 min, and finally decreasing to initial conditions and re-equilibrating the column for 10 min.

The UHPLC system was coupled to a compact™ (Bruker Daltonics) mass spectrometer with an electrospray ionization source. Eluted compounds were detected from *m/z* 50–1200 in negative ion mode. 

Raw data were processed with the software DataAnalysis 4.2 from Bruker Daltonics. Extracted ion chromatograms (EIC) for specific [M−H]^−^ ions were used to locate compounds. The identification of FPCs was based on the UV absorbance at 275 nm, measured [M−H]^−^ with less than ± 2 ppm error when compared to the accurate [M−H]^−^, and the presence of the diagnostic fragment ions *m/z* 249, 207, and 181 observed in the authentic analytical standards (BOCSCI Inc. NY, USA). Calibration curves covered the range 0.5–75 µM and were used for absolute quantification of the six compounds, macrocarpal A, macrocarpal D, macrocarpal J, macrocarpal L, macrocarpal N, and sideroxylonal A corresponding to the analytical standards. The other FPCs detected were labeled as FPC1, FPC3, etc., and further divided into two major groups, macrocarpals and sideroxylonals due to their predominance in *E. globulus* foliage [28]. Total macrocarpals (peaks at *m/z* 485.2544, 489.2857, and 471.2752 include macrocarpals and other FPCs with similar structure, such as euglobals; FPC1 to FPC20 in Table 1) were quantified using the calibration curve for macrocarpal A; while total sideroxylonals (peaks at 499.1609; FPC21 to FPC23 in Table 1) were quantified using the calibration curve for sideroxylonal A. The sum of total macrocarpals and total sideroxylonals were used for the quantification of total FPCs. 

### 4.4. Total Phenolic Contents Extraction and Determination

The total phenolic contents were determined by a Folin-Ciocalteu assay [91,92] with modifications as explained here. To extract the total phenolics contents in eucalypt leaves, 1 mL 50% acetone (acetone/deionized H_2_O, *v*/*v*) was added to 50 mg frozen, homogenized leaf powder on ice and vortexed. After incubating the extraction mixture at room temperature with shaking for 15 min, the mixture was vortexed again and centrifuged at 10000× *g* for 5 min. The supernatant was transferred to new tubes, 1 mL 50% acetone was added to the pellet, and the extraction procedure was repeated twice. The supernatant of the three extractions was combined and stored on ice in darkness. For the Folin-Ciocalteu assay, gallic acid (Sigma-Aldrich GmbH, Germany) was used as the standard. The reaction mixture containing 40 µL standard solution/sample extracts, 1560 µL deionized H_2_O, and 100 µL Folin-Ciocalteu reagent (Sigma-Aldrich GmbH, Germany) was incubated for 8 min at room temperature and 300 µL 20% Na_2_CO_3_ (*w*/*v* in deionized H_2_O) was added to each tube and vortexed. After 2 h incubation at room temperature, the absorbance was determined at 765 nm with a Shimadzu UV2550PC spectrophotometer (Shimadzu, Kyoto, Japan). The total phenolic contents were expressed as gallic acid equivalents (mg of GAE g^−1^ DM) using the standard curve with gallic acid (0 to 1 mg ml^−1^; *r* = 0.99).

### 4.5. Statistical Analysis

In this study, three replicates from different plants were used in all the treatments. The statistical impacts of wounding, O_3_, and O_3_ + wounding combination, and different recovery times on time-dependent changes of the concentration of individual and total FPCs, total macrocarpals and sideroxylonals, the ratio of individual FPCs to the total FPCs, and the total phenolic contents were analyzed separately by using linear mixed models (SPSS 22.0, Chicago, IL, USA) with treatments (wounding, O_3,_ or wounding+ O_3_) and recovery time as fixed effects. Log10 transformation was used where necessary to satisfy the assumption of the linear mixed model. In all cases, paired comparisons among the levels of the same factor were tested for significance by comparing differences of least squares means, at *P* < 0.05. Principal component analysis (PCA) was used to evaluate the effects of different treatments on the coordinated changes of individual FPCs concentrations across the recovery times. Loading and score plots were initially derived after mean-centering and cube root transformation by MetaboAnalyst version 3.0 [93,94] and then redrawn in OriginLab 8.0 (OriginLab Corporation, Northampton, MA, USA).

## 5. Conclusions

In this study, O_3_ and its combination with wounding were shown to influence the abundance of FPCs, resulting in temporal variations of specific compounds in the leaves of the ecologically and economically important tree *E. globulus*. These novel findings open the debate for different roles of this class of specialized metabolite in abiotic stresses, besides the defense against herbivores. The two predominant FPC classes in *E. globulus* leaves, macrocarpals and sideroxylonals, exhibited different response patterns to single and combined treatments of O_3_ and wounding. The single O_3_ treatments induced increased accumulation in individual and total macrocarpals but not in sideroxylonal A and total sideroxylonals; while the single wounding treatment had no effects on the total macrocarpals but resulted in changes in concentrations of several individual macrocarpals, sideroxylonal A, and total sideroxylonals. Combined O_3_ and wounding treatments only led to changes in total sideroxylonals and some individual FPCs but did not affect the concentration of total macrocarpals and total FPCs in *E. globulus* leaves. In particular, among the six quantified individual FPCs, the concentration of macrocarpal J decreased in all O_3_ and wounding treated leaves. A positive correlation between total FPCs and total phenolic contents and negative correlations between total FPCs and terpenoid emissions from stressed *E. globulus* leaves were observed. Changes in the concentration and composition of FPCs suggest their multiple roles in plant resistance mechanisms to environmental stresses. We presume that the group of sideroxylonals is of great importance in wounding stress, not only caused by herbivore feeding but also by other abiotically mechanical damages, while macrocarpals might play multiple roles such as antioxidants induced by both wounding and O_3_ treatments. Given the negative correlations between total FPC concentrations and terpenoid emissions, syntheses of FPC and terpenoid may be closely connected by competing for the same precursors that exist in terpenoid synthesis pathways.

## Figures and Tables

**Table 1:** Peak assignments of formylated phloroglucinol compounds (FPCs) detected in *E. globulus* leaves as analyzed by UHPLC-DAD-ESI-Q-TOF-MS/MS. Peak numbers shown in the table are presented as in Figure 2.

Peak #	RT [min]	Relative Peak Area%	Calculated [M−H]^−^ *m*/*z*	Measured [M−H]^−^ *m*/*z*	Error (ppm)	MS/MS (Relative Intensity, in %)	Molecular Formula	Identity
1	8.9	1.2	489.2857	489.2866	1.8	**489 (100)**, 490 (35), 491 (8)	C_28_H_42_O_7_	FPC01
2	9.6	0.5	489.2857	489.2864	1.4	**489 (100)**, 490 (28), 491 (5)	C_28_H_42_O_7_	**FPC02 Macrocarpal J**
3	10.2	0.1	485.2544	485.2543	−0.2	**485 (100)**, 486 (41), 487 (14), 249 (8)	C_28_H_38_O_7_	FPC03
4	11.1	0.2	485.2544	485.2547	0.6	**485 (100)**, 486 (28), 487 (15), 249 (10)	C_28_H_38_O_7_	FPC04
5	11.3	0.9	485.2544	485.2550	1.2	**485 (100)**, 486 (23), 207 (40), 250 (13)	C_28_H_38_O_7_	FPC05
6	11.6	0.9	485.2544	485.2549	1.0	**485 (100)**, 486 (26), 487 (7), 207 (5)	C_28_H_38_O_7_	FPC06
7	11.8	1.2	485.2544	485.2542	−0.4	**485 (100)**, 486 (23), 207 (28), 250 (15)	C_28_H_38_O_7_	FPC07
8	12.8	0.4	485.2544	485.2547	0.6	**485 (100)**, 486 (23), 487 (10), 207 (3)	C_28_H_38_O_7_	FPC08
9	13.1	0.4	485.2544	485.2550	1.2	**485 (100)**, 486 (40), 487 (8), 207 (10)	C_28_H_38_O_7_	FPC09
10	14.5	1.7	485.2544	485.2546	0.4	**485 (100)**, 486 (29), 207 (9), 250 (10)	C_28_H_38_O_7_	**FPC10 Macrocarpal N**
11	14.7	2.1	471.2752	471.2756	0.8	**471 (100)**, 472 (32), 473 (4)	C_28_H_40_O_6_	FPC11
12	15.3	15.2	471.2752	471.2751	−0.2	**471 (100)**, 472 (27), 473 (4), 207 (3)	C_28_H_40_O_6_	**FPC12 Macrocarpal A**
13	15.6	6.9	471.2752	471.2751	−0.2	**471 (100)**, 472 (28), 473 (5), 207 (3)	C_28_H_40_O_6_	FPC13
14	15.9	7.7	485.2544	485.2545	0.2	**485 (100)**, 486 (29), 207 (8), 250 (2)	C_28_H_38_O_7_	FPC14
15	16.3	16.2	471.2752	471.2751	−0.2	**471 (100)**, 472 (27), 473 (4), 207 (3)	C_28_H_40_O_6_	FPC15
16	16.9	15.8	471.2752	471.2752	0.0	**471 (100)**, 472 (29), 473 (5), 207 (3)	C_28_H_40_O_6_	FPC16
17	17.1	0.5	485.2544	485.2544	0.0	**485 (100)**, 486 (30), 207 (12), 250 (2)	C_28_H_38_O_7_	FPC17
18	17.8	33.1	471.2752	471.2750	−0.4	**471 (100)**, 472 (27), 473 (4), 207 (2)	C_28_H_40_O_6_	**FPC18 Macrocarpal L**
19	18.2	24.2	471.2752	471.2751	−0.2	**471 (100)**, 472 (27), 473 (4), 207 (4)	C_28_H_40_O_6_	FPC19
20	19.0	49.2	471.2752	471.2750	−0.4	**471 (100)**, 207 (48), 250 (28), 472 (26)	C_28_H_40_O_6_	**FPC20 Macrocarpal D**
21	22.3	15.7	499.1609	499.1608	−0.2	**249 (100)**, 250 (15), 181 (1), 251 (1)	C_26_H_28_O_10_	FPC21
22	23.3	18.3	499.1609	499.1610	0.2	**249 (100)**, 250 (13), 181 (1), 251 (2)	C_26_H_28_O_10_	FPC22
23	23.8	100.0	499.1609	499.1607	−0.4	**249 (100)**, 250 (12), 181 (3), 251 (1)	C_26_H_28_O_10_	**FPC23 Sideroxylonal A**

**Table 2:** Summary of the linear mixed model for the statistical effects of treatments (wounding, O_3_, and O_3_ + wounding) and different recovery times on the concentration of individual FPCs in *E. globulus* leaves. Significant values are shown in italic and bold (*n* = 3, *Sig.* < 0.05).

Concentration (mg g^−1^ DM)	Macrocarpal A		Macrocarpal D		Macrocarpal J		Macrocarpal L		Macrocarpal N		Sideroxylonal A	
	*F*	*Sig.*	*F*	*Sig.*	*F*	*Sig.*	*F*	*Sig.*	*F*	*Sig.*	*F*	*Sig.*
Wounding	0.04	0.84	0.96	0.37	N.A.^1^	N.A.^1^	62.43	***0.00***	0.51	0.50	35.32	***0.00***
Time	0.27	0.62	0.02	0.88	N.A.^1^	N.A.^1^	0.09	0.78	1.11	0.33	0.00	0.95
Time × Time	0.30	0.60	0.02	0.89	N.A.^1^	N.A.^1^	0.07	0.81	3.91	0.10	0.28	0.62
Wounding × Time	0.00	0.97	5.49	0.06	N.A.^1^	N.A.^1^	2006.52	***0.00***	10.34	***0.02***	12.71	***0.01***
Ozone	48.01	***0.00***	88.77	***0.00***	1.84	0.22	721.19	***0.00***	41.56	***0.00***	46.06	***0.00***
Time	10.78	***0.02***	2.48	0.17	0.56	0.48	2.68	0.16	2.39	0.17	0.08	0.78
Time × Time	5.27	0.06	1.52	0.26	0.39	0.55	1.75	0.23	4.19	0.09	0.40	0.55
Ozone × Time	18.30	***0.01***	2.96	0.14	13.97	***0.01***	21.69	***0.00***	41.69	***0.00***	17.51	***0.01***
Ozone + Wounding	6.22	***0.05***	1.07	0.34	4.41	0.08	0.81	0.40	23.26	***0.00***	140.11	***0.00***
Time	0.06	0.82	0.11	0.75	0.11	0.75	0.18	0.69	0.28	0.62	0.03	0.87
Time × Time	0.13	0.73	0.23	0.65	0.20	0.67	0.36	0.57	0.99	0.36	0.00	0.99
(Ozone + Wounding) × Time	20.38	***0.00***	1.01	0.35	3.36	0.12	0.00	0.98	5.76	0.05	14.72	***0.01***

^1^ In all the wounding-treated *E. globulus* leaves across the whole recovery period, the concentration of macrocarpal J is out of the detection limit and considered as zero. Therefore, the effects of wounding on macrocarpal J are not analyzed by the linear mixed model. N.A., not statistically analyzed.

**Table 3:** Summary of the linear mixed model for the statistical effects of treatments (wounding, O_3_, and O_3_ + wounding) and different recovery times on total macrocarpals, total sideroxylonals, total FPCs, and total phenolic contents in *E. globulus* leaves. Significant values are shown in italic and bold (*n* = 3, *Sig.* < 0.05).

Concentration (mg g^−1^ DM)	Total Macrocarpals		Total Sideroxylonals		Total FPCs		Total Phenolics	
	*F*	*Sig.*	*F*	*Sig.*	*F*	*Sig.*	*F*	*Sig.*
Wounding	4.42	0.08	27.90	***0.00***	15.70	***0.01***	115.88	***0.00***
Time	0.94	0.37	0.42	0.54	0.67	0.44	0.40	0.56
Time × Time	1.04	0.35	0.65	0.45	0.87	0.39	2.17	0.19
Wounding × Time	0.00	0.98	13.71	***0.01***	1.72	0.24	27.52	***0.00***
Ozone	20.56	***0.00***	5.33	0.06	64.02	***0.00***	12.30	***0.01***
Time	1.51	0.27	0.01	0.94	2.80	0.15	4.31	0.08
Time × Time	4.75	0.07	0.26	0.63	5.33	0.06	6.55	***0.04***
Ozone × Time	0.09	0.77	2.56	0.16	0.54	0.49	10.47	***0.02***
Ozone + Wounding	0.01	0.94	16.44	***0.01***	1.45	0.27	924.63	***0.00***
Time	1.99	0.21	0.04	0.85	1.29	0.30	0.07	0.79
Time × Time	4.65	0.07	0.29	0.61	2.78	0.15	0.39	0.55
(Ozone + Wounding) × Time	0.28	0.62	2.79	0.15	1.50	0.27	176.34	***0.00***

**Table 4:** Summary of the linear mixed model for the statistical effects of treatments (wounding, O_3_, and O_3_ + wounding) and different recovery times on the composition of individual FPCs in total FPCs in *E. globulus* leaves. Significant values are shown in italic and bold (*n* = 3, *Sig.* < 0.05).

Composition (%)	Macrocarpal A		Macrocarpal D		Macrocarpal J		Macrocarpal L		Macrocarpal N		Sideroxylonal A	
	*F*	*Sig.*	*F*	*Sig.*	*F*	*Sig.*	*F*	*Sig.*	*F*	*Sig.*	*F*	*Sig.*
Wounding	13.53	***0.01***	29.27	***0.00***	N.A.^1^	N.A.^1^	16.96	***0.01***	4.81	0.07	302.54	***0.00***
Time	7.13	***0.04***	5.43	0.06	N.A.^1^	N.A.^1^	4.32	0.08	0.97	0.36	0.07	0.81
Time × Time	4.50	0.08	6.04	0.05	N.A.^1^	N.A.^1^	4.73	0.07	0.04	0.85	0.20	0.67
Wounding × Time	1.72	0.24	15.94	***0.01***	N.A.^1^	N.A.^1^	5.33	0.06	29.86	***0.00***	131.52	***0.00***
Ozone	12.74	***0.01***	1.18	0.32	106.17	***0.00***	0.51	0.50	0.23	0.65	39.21	***0.00***
Time	1.19	0.32	4.19	0.09	2.74	0.15	2.04	0.20	3.22	0.13	0.39	0.55
Time × Time	0.40	0.55	2.10	0.20	1.54	0.26	1.61	0.25	4.45	0.08	2.83	0.14
Ozone × Time	7.78	***0.03***	12.09	***0.01***	0.13	0.73	4.83	0.07	7.98	***0.03***	13.54	***0.01***
Ozone + Wounding	22.29	***0.00***	0.89	0.38	47.33	***0.00***	4.23	0.09	7.10	***0.04***	16.99	***0.01***
Time	2.10	0.20	0.10	0.77	0.17	0.69	0.01	0.91	35.06	***0.01***	0.00	0.95
Time ×* Time	1.14	0.33	0.25	0.64	0.02	0.90	0.05	0.83	67.31	***0.00***	0.29	0.61
(Ozone + Wounding) × Time	88.99	***0.00***	0.48	0.52	2.27	0.18	4.11	0.09	2.21	0.19	160.49	***0.00***

^1^ In all the wounding-treated *E. globulus* leaves across the whole recovery period, the concentration of macrocarpal J is out of the detection limit and considered as zero. The composition of macrocarpal J in total FPCs of wounding treated leaves is zero accordingly. Therefore, the effects of wounding on macrocarpal J are not analyzed by the linear mixed model. N.A., not statistically analyzed.

**Table 5:** Effects of treatments (control, wounding, O_3_, O_3_ and wounding treatments) on the ratio of each individual FPC to the total FPCs in *E. globulus* leaves. Data shown are means ± SE (*n* = 3). Details of the statistical summary are shown in Table 4. C, control; W, wounding; O, O_3_ and O + W, O_3_ + wounding.

% of Total FPCs		Macrocarpal A	Macrocarpal D	Macrocarpal J	Macrocarpal L	Macrocarpal N	Sideroxylonal A
**0.5**	**C**	1.42 ± 0.82	7.61 ± 4.39	0.06 ± 0.03	9.03 ± 5.21	0.36 ± 0.21	29.31 ± 16.92
	**W**	1.02 ± 0.59	4.05 ± 2.34	0.00 ± 0.00	4.92 ± 2.84	0.39 ± 0.22	23.82 ± 13.75
	**O**	2.13 ± 1.23	6.52 ± 3.76	0.00 ± 0.00	7.97 ± 4.60	0.17 ± 0.10	15.88 ± 9.17
	**O + W**	0.85 ± 0.49	4.58 ± 2.64	0.02 ± 0.01	6.31 ± 3.64	0.14 ± 0.08	20.07 ± 11.58
**25**	**C**	1.27 ± 0.73	5.54 ± 3.20	0.07 ± 0.04	6.43 ± 3.71	0.29 ± 0.17	22.80 ± 13.16
	**W**	0.90 ± 0.52	3.59 ± 2.07	0.00 ± 0.00	4.63 ± 2.67	0.36 ± 0.21	30.04 ± 17.34
	**O**	2.68 ± 1.55	7.25 ± 4.18	0.00 ± 0.00	8.26 ± 4.77	0.38 ± 0.22	16.44 ± 9.49
	**O + W**	2.37 ± 1.37	7.62 ± 4.40	0.00 ± 0.00	9.49 ± 5.48	0.45 ± 0.26	20.23 ± 11.68
**50**	**C**	1.10 ± 0.63	4.70 ± 2.71	0.08 ± 0.04	6.68 ± 3.85	0.11 ± 0.06	27.45 ± 15.84
	**W**	1.10 ± 0.64	4.67 ± 2.70	0.00 ± 0.00	5.54 ± 3.20	0.37 ± 0.21	18.52 ± 10.69
	**O**	1.67 ± 0.96	5.89 ± 3.40	0.00 ± 0.00	8.17 ± 4.71	0.09 ± 0.05	19.35 ± 11.17
	**O + W**	1.84 ± 1.06	7.07 ± 4.08	0.01 ± 0.01	8.27 ± 4.77	0.38 ± 0.22	22.63 ± 13.06
**75**	**C**	1.49 ± 0.86	6.09 ± 3.51	0.09 ± 0.05	8.43 ± 4.87	0.18 ± 0.10	21.49 ± 12.41
	**W**	1.19 ± 0.69	5.61 ± 3.24	0.00 ± 0.00	6.14 ± 3.54	0.42 ± 0.24	18.88 ± 10.90
	**O**	1.72 ± 0.99	7.34 ± 4.24	0.00 ± 0.00	8.60 ± 4.96	0.28 ± 0.16	18.31 ± 10.57
	**O + W**	0.92 ± 0.53	4.87 ± 2.81	0.01 ± 0.01	5.87 ± 3.39	0.13 ± 0.07	20.70 ± 11.95

## Supplementary Materials

Figure S1. Correlations between total FPC concentrations and isoprene emissions from wounding, O_3_, and O_3_ + wounding treated *E. globulus* leaves. Figure S2. Correlations between total FPC concentrations and monoterpene emissions from wounding, O_3_, and O_3_ + wounding treated *E. globulus* leaves. Figure S3. Correlations between total FPC concentrations and sesquiterpene emissions from wounding, O_3_, and O_3_ + wounding treated *E. globulus* leaves.
