# Supplementary material for: Ozone and Wounding Stresses Differently Alter the Temporal Variation in Formylated Phloroglucinols in Eucalyptus globulus Leaves
Source: Metabolites. 2019 Mar 6;9(3):46. doi: 10.3390/metabo9030046 (PMC6468632; doi:10.3390/metabo9030046)

Figure S1. Correlations between total FPC concentrations and isoprene emissions (See Kanagendran, *et al.* [7] for graphical representation for isoprene emission) from wounding, O_3 and O_3 + wounding treated *E. globulus* leaves. Each date dot stands for mean of three independent biological replicates from 0.5 h, 25 h, 50 h and 75 h after the treatments.

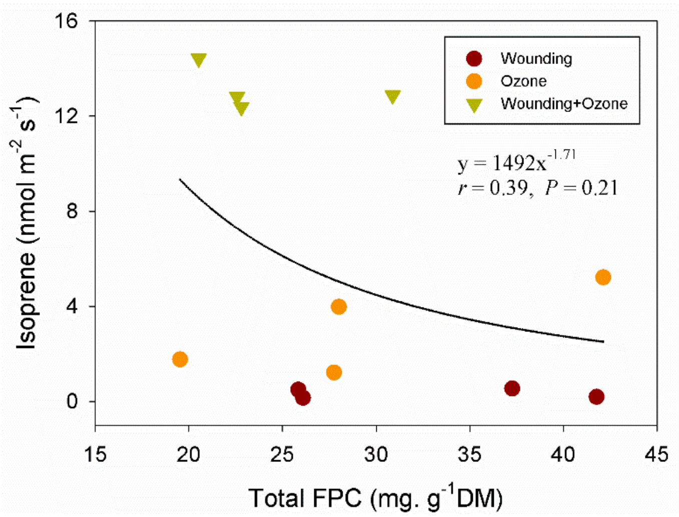

Figure S2. Correlations between total FPC concentrations and monoterpene emissions from wounding, O_3 and O_3 + wounding treated *E. globulus* leaves. Emission rates of three most abundant monoterpenes and the total monoterpenes were used for this correlation (See Kanagendran, *et al.* [7] for graphical representation for monoterpene emission). Each date dot stands for mean of three independent biological replicates from 0.5 h, 25 h, 50 h and 75 h after the treatments.

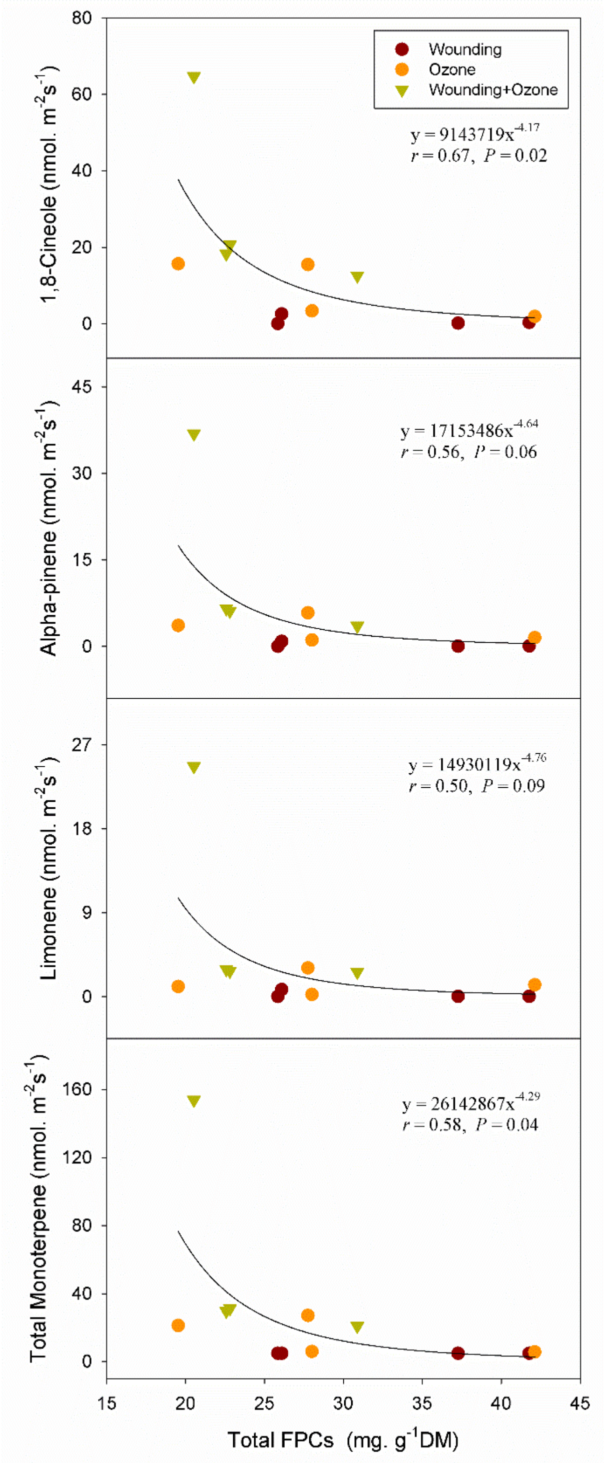

Figure S3. Correlations between total FPC concentrations and sesquiterpene emissions from wounding, O_3 and O_3 + wounding treated *E. globulus* leaves. Emission rates of three most abundant sesquiterpenes and total sesquiterpene (see Kanagendran, *et al.* [7] for graphical representation for sesquiterpene emission) were used for this correlation. Each date dot stands for mean of three independent biological replicates from 0.5 h, 25 h, 50 h and 75 h after the treatments. Outliers were indicated in grey ellipse.

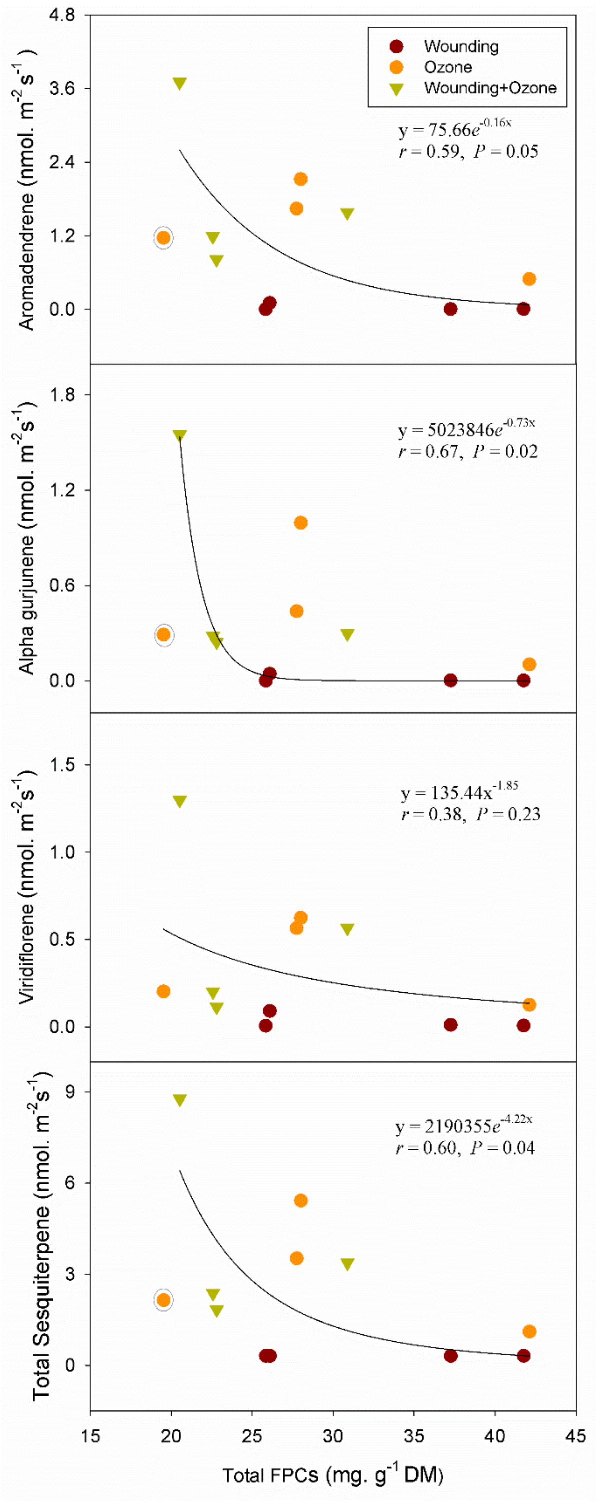

Supplement: Supplementary file 1 [file metabolites-09-00046-s001.pdf]
